# Detection of Microcalcifications in Spiral Breast Computed Tomography with Photon-Counting Detector Is Feasible: A Specimen Study

**DOI:** 10.3390/diagnostics11050848

**Published:** 2021-05-09

**Authors:** Matthias Wetzl, Evelyn Wenkel, Eva Balbach, Ebba Dethlefsen, Arndt Hartmann, Julius Emons, Christiane Kuhl, Matthias W. Beckmann, Michael Uder, Sabine Ohlmeyer

**Affiliations:** 1 Department of Radiology, University Hospital Erlangen, Maximiliansplatz 3, 91054 Erlangen, Germany; evelyn.wenkel@uk-erlangen.de (E.W.); eva.balbach@uk-erlangen.de (E.B.); michael.uder@uk-erlangen.de (M.U.); sabine.ohlmeyer@uk-erlangen.de (S.O.); 2 Department of Diagnostic and Interventional Radiology, University Hospital Aachen, Pauwelsstraße 30, 52074 Aachen, Germany; edethlefsen@ukaachen.de (E.D.); ckuhl@ukaachen.de (C.K.); 3 Department of Pathology, University Hospital Erlangen, Krankenhausstraße 8-10, 91054 Erlangen, Germany; arndt.hartmann@uk-erlangen.de; 4 Department of Gynecology and Obstetrics, University Hospital Erlangen, Universitätsstraße 21/23, 91054 Erlangen, Germany; julius.emons@uk-erlangen.de (J.E.); matthias.beckmann@uk-erlangen.de (M.W.B.)

**Keywords:** spiral breast CT, reconstruction modes, breast cancer, microcalcifications

## Abstract

The primary objective of the study was to compare a spiral breast computed tomography system (SBCT) to digital breast tomosynthesis (DBT) for the detection of microcalcifications (MCs) in breast specimens. The secondary objective was to compare various reconstruction modes in SBCT. In total, 54 breast biopsy specimens were examined with mammography as a standard reference, with DBT, and with a dedicated SBCT containing a photon-counting detector. Three different reconstruction modes were applied for SBCT datasets (Recon1 = voxel size (0.15 mm)^3^, smooth kernel; Recon2 = voxel size (0.05 mm)^3^, smooth kernel; Recon3 = voxel size (0.05 mm)^3^, sharp kernel). Sensitivity and specificity of DBT and SBCT for the detection of suspicious MCs were analyzed, and the McNemar test was used for comparisons. Diagnostic confidence of the two readers (Likert Scale 1 = not confident; 5 = completely confident) was analyzed with ANOVA. Regarding detection of MCs, reader 1 had a higher sensitivity for DBT (94.3%) and Recon2 (94.9%) compared to Recon1 (88.5%; *p* < 0.05), while sensitivity for Recon3 was 92.4%. Respectively, reader 2 had a higher sensitivity for DBT (93.0%), Recon2 (92.4%), and Recon3 (93.0%) compared to Recon1 (86.0%; *p* < 0.05). Specificities ranged from 84.7–94.9% for both readers (*p* > 0.05). The diagnostic confidence of reader 1 was better with SBCT than with DBT (DBT 4.48 ± 0.88, Recon1 4.77 ± 0.66, Recon2 4.89 ± 0.44, and Recon3 4.75 ± 0.72; DBT vs. Recon1/2/3: *p* < 0.05), while reader 2 found no differences. Sensitivity and specificity for the detection of MCs in breast specimens is equal for DBT and SBCT when a small voxel size of (0.05 mm)^3^ is used with an equal or better diagnostic confidence for SBCT compared to DBT.

## 1. Introduction

Microcalcifications are small calcium-containing deposits in breast tissue with a maximum size of 1 mm [1]. Their importance for the detection of malignant breast lesions is well known. In a review of the Swedish screening program, 74% of ductal carcinomas in situ (DCIS) and 30% of invasive breast cancers were associated with microcalcifications [2]. Furthermore, in 32% of cases, microcalcifications are the only imaging feature of invasive breast cancer or DCIS [3].

Four-view full-field digital mammography (FFDM) is the standard examination for the detection and classification of microcalcifications in screening and clinical breast exams [4]. However, 2D imaging modality and superimpositions can decrease sensitivity in dense breast tissue [5,6]. Another method for the detection of MCs is digital breast tomosynthesis (DBT). It is a pseudo-3D method and can improve detectability of soft tissue lesions compared to FFDM [7,8]. Concerning microcalcifications, there are studies that favor DBT over FFDM and the other way around. It is still under discussion if the detection rate and especially morphological features in DBT can be evaluated as good as in FFDM [9,10,11,12]. Breast computed tomography is an imaging modality that enables true three-dimensional imaging without superimpositions of breast tissue structures. In addition, contrast-enhanced imaging is possible, which improves the visualization of malignant breast masses compared to digital mammography [13]. So far, different breast computed tomography systems have been evaluated. Lindfors et al. developed a cone-beam breast-CT, in which visualization of masses was significantly better compared to mammography, but visualization of MCs was significantly worse [14]. Shah et al. constructed a tilt-capable combination of SPECT and cone beam-CT to gain a better sampling of breast tissue close to the chest wall [15]. Evaluation of a commercially available cone-beam breast-CT showed that detection of malignant lesions, including DCIS, was superior compared to mammography, but results regarding the detection of MCs are lacking [16]. Lately developed dedicated breast CTs with photon-counting detectors enable improved spatial resolution at lower radiation doses compared to cone-beam breast-CTs. This is due to decreased detector pixel size and a direct conversion of x-ray photons into electric charge, which decreases intrinsic noise. In a phantom study, Cho et al. concluded that dedicated photon-counting breast CT scanners perform better than cone beam CT and could even improve detection of microcalcifications (MCs) compared to mammography [17]. Kalender et al. developed a high-resolution spiral breast CT (SBCT) with a spiral acquisition mode and a photon-counting detector. In an initial simulation study with the SBCT, microcalcifications up to a size of 0.15 mm and clusters were clearly visible [18]. A following ‘proof of concept’-study of 10 surgical specimen enabled high resolution imaging at a low radiation dose, which was comparable to DBT or screening mammographies [19]. First clinical experiences with non-contrast enhanced SBCT showed a promising detection rate of mass lesions [20,21]. Recommendations regarding optimal examination parameters dependent on breast size and tissue composition were recently published [22]. However, optimal reconstruction parameters are still not established and clinical studies addressing detection of microcalcifications do not exist.

The primary objective of the study was to compare SBCT to DBT for the detection of MCs in breast specimens using sensitivity, specificity, and diagnostic confidence. The secondary objective was to compare sensitivity and specificity of various reconstruction modes in SBCT.

## 2. Materials and Methods

### 2.1. Study Population

Inclusion criterion for patients (mean age 57.7 ± 9.0 years, range 40.1 to 77.8 years) was a planned vacuum-assisted biopsy of the breast due to suspicious microcalcifications in mammography (BI-RADS 4 or 5) [1]. The prospective study was performed from November 2019 to July 2020. In total, 54 consecutive breast specimens from vacuum-assisted biopsies out of daily clinical routines were examined.

### 2.2. Imaging Technique

Biopsy specimens were examined with FFDM, DBT (both at 23 kV, 100 mA; device: Mammomat Inspiration, Siemens Healthcare GmbH, Erlangen, Germany), and with a dedicated spiral breast computed tomography (nu:view, AB-CT–Advanced Breast-CT GmbH, Erlangen, Germany).

**Figure 1:**
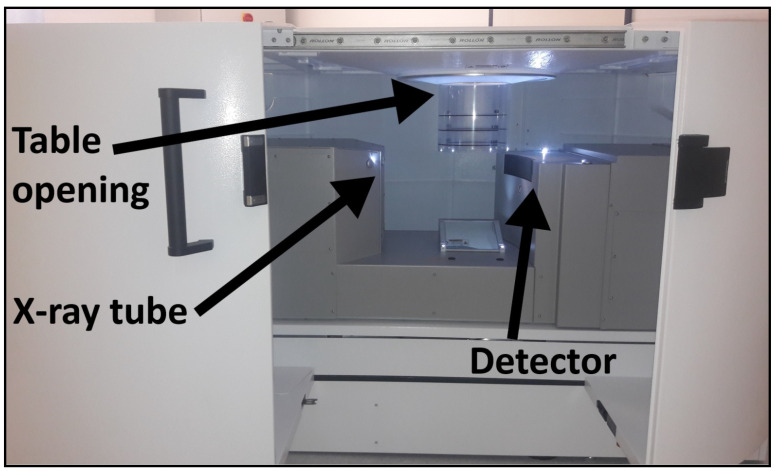
Design of the dedicated spiral breast CT. The patient lies on the patient table in prone position. The concave shape of the patient table around the table opening enables imaging of breast tissue up to the chest wall. The breast hangs freely through the table opening and is covered by a cylindric safety cover. The x-ray tube/detector unit rotates on a spiral path around the breast.

The SBCT scanner was equipped with a cadmium telluride photon-counting detector (AB-CT: Advanced Breast-CT GmbH, in cooperation with Direct Conversion Group, Erlangen, Germany). The detector had a pixel size of (0.1 mm)^2^ and converted x-ray photons directly into electric charge. X-ray generation was performed at a fixed tube voltage of 60 kV and a focal spot size of 0.3. The beam was prefiltered by 3 mm aluminum. Scans were acquired with a pitch factor of 1 and a tube current of 50 mA. Raw data were acquired in single-energy mode since software for energy discrimination of photons was not available for the SBCT. Figure 1 shows the design of the SBCT.

### 2.3. Image Reconstruction of SBCT and DBT

Raw data of the SBCT were reconstructed using Feldkamp-type filtered back-projection (FBP) with three different parameter sets:

- Recon1: voxel size of (0.15 mm)^3^, smooth kernel, 2 × 2 detector binning, reconstruction time of 8 min.
- Recon2: voxel size of (0.05 mm)^3^, smooth kernel, 1 × 1 detector binning, reconstruction time of 15 min.
- Recon3: voxel size of (0.05 mm)^3^, sharp kernel, 1 × 1 detector binning, reconstruction time of 15 min.

Recon2 and Recon3 were experimental reconstruction sets.

For the DBT system, a calcium-sensitive reconstruction algorithm of the DBT system called ‘standard calcium diagnosis’ was used for image interpretation. In a sub-study of five patients, DBT with this reconstruction mode was best for the detection of MCs compared to two other DBT-reconstructions offered by the vendor.

### 2.4. Reference Standard

**Figure 2:**
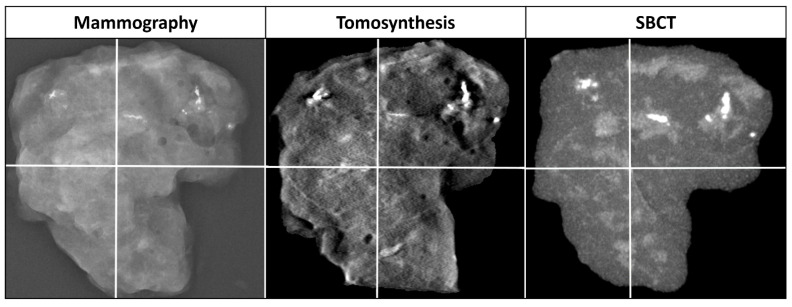
Division of a specimen into four quadrants in mammography, digital breast tomosynthesis, and spiral breast-CT (SBCT). Both readers evaluated specimens per quadrant. In this case, SBCT with a Recon1 reconstruction set and maximum intensity projection (slice thickness 4 mm) is displayed to visualize microcalcifications of different depths in one image. Depiction of details is clearer with thinner slice thicknesses.

**Figure 3:**
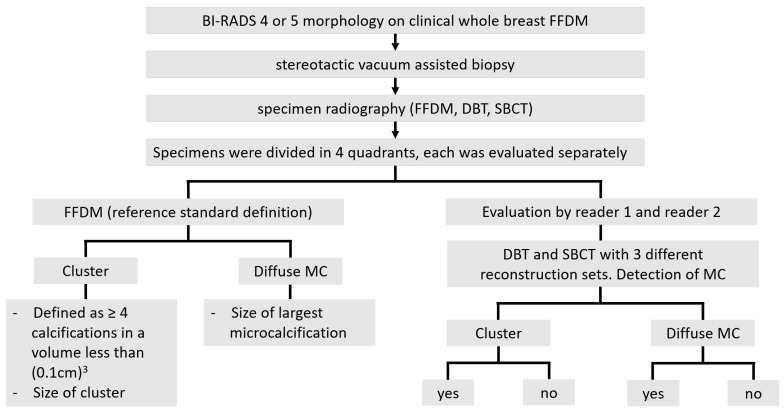
Study workflow. A study coordinator defined microcalcifications in FFDM and classified them as either clusters or diffuse distributed (left side). The two blinded readers evaluated DBT and SBCT reconstructions (right side). Microcalcifications (MCs), full-field digital mammography (FFDM), digital breast tomosynthesis (DBT), and spiral breast computed tomography (SBCT).

FFDM of specimens was used to establish a reference standard, since superimpositions were not expected in biopsy specimens and the high resolution of FFDM allowed the detection of all radiographically detectable microcalcifications. Each image of a biopsy specimen was divided into 4 quadrants and images were always analyzed per quadrant (Figure 2). Since MCs were not present in each quadrant, analysis included quadrants with and without MCs. A study coordinator evaluated each quadrant in FFDM for the presence of MCs, type of MC (cluster, diffuse), maximum diameter of diffuse microcalcifications within a quadrant, and long and short diameter of a whole cluster (Figure 3). For this study, a cluster was individually defined as ≥ 4 microcalcifications within an area less than (0.1 cm)^2^. This definition of a cluster appeared to be useful in this experimental setting for distinguishing between singular and grouped microcalcifications, since grouped microcalcifications are associated with a higher rate of precancerous lesions [23]. Volumetric size of the biopsy specimens was determined with a dedicated software (Syngo.via VA40A, Siemens Healthcare GmbH, Erlangen, Germany).

### 2.5. Image Analysis

Two blinded readers, with 15 and 10 years of experience in reading FFDM and DBT evaluated biopsy specimens, evaluated each quadrant for the detection of microcalcifications with three SBCT reconstruction sets and DBT. Furthermore, the distribution of MCs was analyzed (cluster or diffuse; Figure 3). SBCT reconstructions were evaluated with maximum intensity projection (MIP), 2 mm slice thickness with increments of 0.05 mm or 0.15 mm, dependent on reconstruction mode, and a default window setting with a center of 600 HU and a width of 2000 HU. Our clinical experience showed the best detectability of MCs with these settings. However, readers were able to adjust window settings and change into multiplanar reconstruction mode (MPR) at their discretion. Window settings of DBT could also be adjusted. Additionally, the blinded readers rated their diagnostic confidence for the detectability of microcalcifications on a 5-point Likert scale (1 not confident, 2 slightly confident, 3 moderately confident, 4 highly confident, 5 completely confident).

### 2.6. Statistical Analysis

Statistical analysis was performed using Excel 365 (Microsoft, Redmond, DC, USA), SPSS software version 24 (IBM, Armonk, NY, USA), and vassarstats.net (Richard Lowry, Vassar College, Poughkeepsie, NY, USA). Sensitivities and specificities were calculated for DBT and SBCT reconstructions. Furthermore, the correct classification as a cluster and the correct number of diffuse distributed MCs were evaluated. A sub-analysis of small MCs (maximum diameter ≤ 0.05 mm) was performed, as well. Confidence intervals were calculated for both readers, according to Newcombe [24]. The McNemar test was used to compare the diagnostic performance of DBT and SBCT reconstructions for both readers. For multiple comparisons of diagnostic confidence, the one-way analysis of variance (ANOVA) multiple comparison test with post hoc pairwise comparisons (Bonferroni or Games–Howell) was applied. All tests were performed two-sided, and *p* < 0.05 was considered to be statistically significant. Inter-rater agreement was evaluated by using Cohen’s Kappa value (κ). κ was interpreted as follows: 0.6 < κ ≤ 0.8 = substantial agreement, 0.8 < κ ≤ 1.0 = almost perfect agreement.

## 3. Results

### 3.1. Reference Standard

In total, 54 biopsy specimens (39 benign, 15 malignant) were divided into 216 segments, in which microcalcifications were present in 72.7% (only clusters in 14.4%, only diffuse MCs in 36.1%, and cluster and diffuse MCs in 22.2%). The mean volume of the analyzed biopsy specimens was 2.3 ± 0.8 cm^3^.

### 3.2. Detection of Microcalcifications

Reader 1 showed sensitivities and specificities of 94.3/84.7% for DBT, 88.5/94.9% for Recon1, 94.9/91.5% for Recon2, and 92.4/93.2% for Recon3. Compared to Recon1, sensitivity was higher for DBT (*p* = 0.049) and Recon2 (*p* = 0.006), while all other sensitivities and specificities did not differ significantly. 

Respectively, sensitivities and specificities of reader 2 were 93.0/91.5% for DBT, 86.0/94.9% for Recon1, 92.4/93.2% for Recon2, and 93.0/91.5% for Recon3. Sensitivity for Recon1 was lower compared the other groups (Recon1 vs. DBT: *p* = 0.027; Recon1 vs. Recon2: *p* = 0.041; Recon1 vs. Recon3: *p* = 0.027), while all other sensitivities and specificities did not differ significantly. Levels of significance are shown in Table 1. 

**Figure 4:**
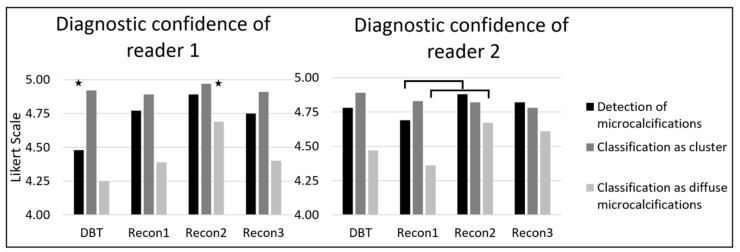
The diagnostic confidence of readers 1 and 2 on a 5-point Likert scale. Comparison of digital breast tomosynthesis (DBT) and three different reconstructions of the dedicated spiral breast computed tomography (SBCT; Recon1 = voxel size of (0.15 mm)^3^, smooth kernel; Recon2: voxel size of (0.05 mm)^3^, smooth kernel; Recon3: voxel size of (0.05 mm)^3^, sharp kernel). Diagnostic confidence for the detection of microcalcifications was best for Recon2. Diagnostic confidence for the classification as a cluster was equal in all groups. Diffuse microcalcifications were easiest classified with SBCT, especially with Recon2. Significant differences (*p* < 0.05) between two groups are marked with a line. The differences between one group (star) and the other three groups are also marked.

The diagnostic confidence of reader 1 for the detection of microcalcifications was better for SBCT than DBT (4.48 ± 0.88 for DBT, 4.77 ± 0.66 for Recon1, 4.89 ± 0.44 for Recon2 and 4.75 ± 0.72 for Recon3; DBT vs. Recon1/2/3: *p* < 0.05, all other *p* > 0.05). The diagnostic confidence of reader 2 was worst for Recon1 and best for Recon2 (DBT 4.78 ± 0.68, Recon1 4.69 ± 0.70, Recon2 4.88 ± 0.42, and Recon3 4.82 ± 0.60; Recon1 vs. Recon2: *p* < 0.006, all other *p* > 0.05). An overview of diagnostic confidences is shown in Figure 4.

The inter-rater agreement was substantial for DBT (κ = 0.79) and almost perfect for SBCT (Recon1: κ = 0.86; Recon2: κ = 0.88; Recon3: κ = 0.89).

### 3.3. Classification as Cluster

**Figure 5:**
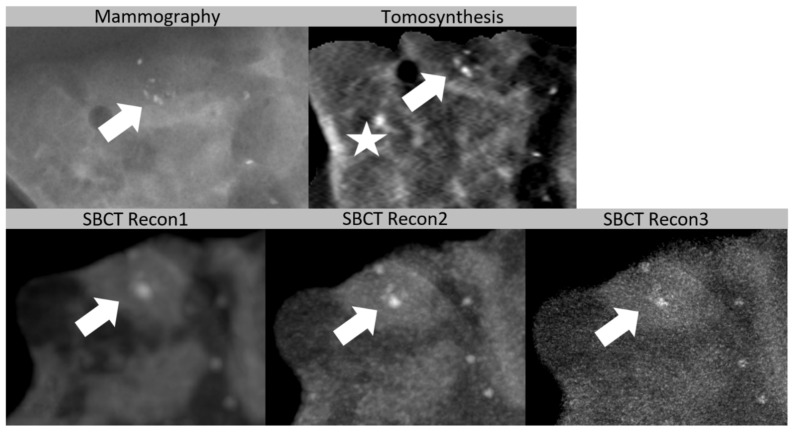
Cluster of microcalcifications in a biopsy specimen of a 58-year-old patient. Histopathology was benign (sclerosing adenosis). The cluster (arrow) can be clearly recognized in spiral breast computed tomography (SBCT) and in tomosynthesis. In this case, differentiation was best with Recon2 (voxel size (0.05 mm)^3^, smooth kernel), compared to tomosynthesis and Recon1 (voxel size (0.15 mm)^3^, smooth kernel) or Recon3 (voxel size (0.05 mm)^3^, sharp kernel). Diffuse microcalcifications are clearly visible with tomosynthesis and SBCT. Tomosynthesis shows a possibly false positive microcalcification (star).

**Figure 6:**
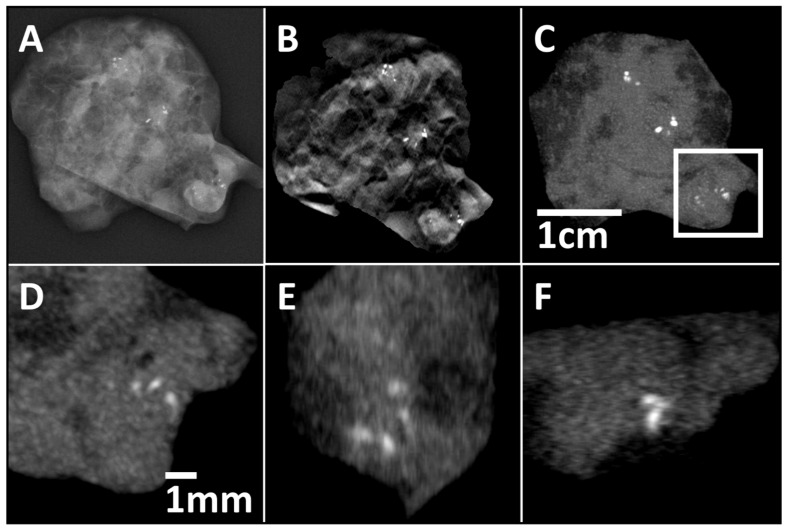
Three different clusters in one specimen can be identified with mammography (**A**), DBT (**B**), and SBCT (Recon2, MIP-reconstruction) with 4 mm slice thickness (**C**). Axial (**D**), sagittal (**E**), and coronar (**F**) MIP-reconstructions with 0.4 mm slice thickness of the cluster in the right lower corner. Details become clearer with thinner slices. A measurement scale for the whole specimen (**A**–**C**) is inserted in (**C**), and a measurement scale for the cluster in the right lower corner (**D**–**F**) is inserted in (**D**).

Clusters were identified in 79 different quadrants (mean long diameter of clusters 2.3 ± 1.0 mm, mean short diameter 1.5 ± 0.7 mm). Figure 5 shows an example of a cluster. Reader 1 correctly identified clusters in 79.7% with DBT, in 54.4% with Recon1, in 62.0% with Recon2, and in 59.5% with Recon3, with a significant difference between DBT and Recon1/2/3 (*p* < 0.05). Reader 2 identified clusters correctly in 77.2% with DBT, in 62.0% with Recon1, in 75.9% with Recon2, and in 75.9% with Recon3, with a significant difference between Recon 1 and DBT/ Recon2/3 (*p* < 0.05). A complete list of values for each group is shown in Table 2. Further analysis of clusters is feasible in SBCT with multiplanar reconstructions (Figure 6).

Diagnostic confidence of cluster identification was high in all imaging modalities, with Likert scores between 4.89 and 4.97 for reader 1 (ANOVA: *p* = 0.38) and between 4.78 and 4.97 for reader 2 (ANOVA: *p* = 0.42; Figure 4).

### 3.4. Classification as Diffuse Microcalcification

Regarding the detection of diffuse microcalcifications, neither of the two readers found significant differences between DBT, Recon2, or Recon 3 (Table 2). For reader 1, there was a significant difference between Recon1 (81%) and Recon2/Recon3 (92.1/88.9%; Recon1 vs. Recon 2: *p* = 0.001; Recon1 vs. Recon3: *p* = 0.021). Respective values for reader 2 were 80.2% for Recon1 and 84.1–88.1% for DBT and Recon2/3 (*p* = 0.170). 

When focusing on small microcalcifications with a maximum diameter of 0.5 mm, detection rates of diffuse microcalcifications were still high in all groups (Table 2). Respective values for reader 1/2 were 86.3/85.3% for DBT, 76.5/78.4% for Recon1, 90.2/82.4% for Recon2, and 86.3/80.4% for Recon3. Reader 1 performed significantly better with Recon2/3 than with Recon1 (Recon1 vs. Recon2: *p* = 0.001; Recon1 vs. Recon3: *p* = 0.021), while reader 2 found no significant differences in detection rates of small diffuse microcalcifications.

The diagnostic confidence of diffuse microcalcification classification for both readers was best with Recon2 (reader 1: 4.25 ± 0.92 for DBT, 4.39 ± 0.94 for Recon1, 4.69 ± 0.69 for Recon2, and 4.40 ± 1.01 for Recon3, Recon2 vs. DBT/Recon1/3: *p* < 0.05; reader 2: 4.47 ± 0.94, 4.36 ± 0.88, 4.67 ± 0.66, and 4.61 ± 0.76, Recon1 vs. Recon2: *p* < 0.05 respectively; Figure 4).

## 4. Discussion

This is the first study that has systematically evaluated the detection of MCs in specimens with a clinical SBCT system. Furthermore, no other study has compared SBCT with DBT for the detection of MCs so far. The study of the specimens showed that, with SBCT, detection of MCs is feasible with a high sensitivity and specificity and on a high level of diagnostic confidence. SBCT with small voxel sizes (0.05 mm)^3^ performed as good as DBT for the detection of MCs. The detection of MCs in SBCT was better with small voxel sizes of (0.05 mm)^3^ than with voxel sizes of (0.15 mm)^3^. Nevertheless, a voxel size of (0.15 mm)^3^ still enabled high detection rates of MCs, with a sensitivity between 86.0–88.5% and a specificity of 94.9%. 

Concerning the correct classification as a cluster, reader 1 was significantly better with DBT compared to Recon1/2/3 and reader 2 was significantly better with DBT/ Recon2/3 compared to Recon1. Therefore, a limited distinction of different microcalcifications within a cluster in the SBCT reconstruction set with a voxel size of (0.15 mm)^3^ could be one reason for this finding. The almost perfect inter-rater agreement for SBCT, which was better compared to DBT, and the high diagnostic confidence of SBCT showed the potential of SBCT to visualize MCs. Detection of diffuse MCs was high for all study groups, even for small MCs less than or equal to 0.5 mm. Rößler et al. evaluated the detection of microcalcifications in 30 specimens with FFDM, DBT, and SBCT [25]. They also found good sensitivity of SBCT of 85%, compared to 72% with DBT and 82% with FFDM. Shim et al. evaluated clusters of MCs of different sizes with the SBCT in a phantom study. Clusters with MCs of 0.2 mm were detected at a tube current higher than or equal to 25 mA [26]. This supports the high detection rate of small MCs below 0.5 mm in our study. While the potential of MC detection is shown in this study, its characterization is also important. Although this was not part of our study, the high resolution of SBCT with voxel sizes as small as (0.05 mm)^3^ might facilitate the characterization of MCs based on artificial intelligence. This should be analyzed in future studies.

No other studies evaluating microcalcifications in this new SBCT system have been performed to date. However, several studies evaluated detectability of microcalcifications with DBT and cone-beam breast CT. Tagliafico et al. analyzed 107 microcalcifications with FFDM and DBT. The sensitivity of DBT was comparable to our study (91.1%), whereas specificity was 100% [27]. In a study by Spangler et al., detection of microcalcification was better with FFDM (84%) than with DBT (75%) [10]. These results match up with our study. O’Connell et al. detected 84.8% of MCs up to 1 mm diameter with a cone-beam breast CT [28]. These results are not comparable to ours, since it was an in vivo study and diameter of MCs in our study were much smaller (maximum diameter of diffuse MCs was smaller than 0.5 mm in 81%). Lindfors et al. missed 4 (2 benign and 2 malignant) out of 12 microcalcifications with a cone-beam breast CT. Comparisons to our results are limited as well, since this was an in vivo study and average lesion size was 1.5 cm [14]. Our study has some limitations. First, the readers’ experience for the evaluation of microcalcifications in SBCT was limited, since this device was lately introduced to our department. Prior to the evaluation, the readers had training with about 40 cases only. This could result in a bias favoring DBT, where readers have had years of experience. 

Second, our standard reference was mammography, and a comparison with histopathological detected microcalcifications was only feasible based on the written pathological report. Since sizes of specimens were small, we suppose that there was no relevant superimposition and that we detected all radiographically possibly detectable MCs with mammography.

Third, dose measurements for the specimens was not taken into account. While assessment of patient dose is an important aspect for clinical in vivo applications in mammography, tomosynthesis, and breast CT, this study focused on the detection of microcalcifications in breast specimens. Reasonable dose estimation of breast specimens is not given, since it represents only a small part of the complete breast. Specimens differ in shape and tissue density from the complete breast, and certain tissues are not present at all (e.g., skin). Patient dose in breast CT was already investigated in other studies [19,20,22,28,29], and thus, a potential dose delivered to patients when using tube current from this study can be estimated by linear scaling. In addition, the scan parameters used in this study were chosen to determine the system resolution characteristics to produce quasi-noise free images (both in SBCT and DBT), and therefore, a higher tube current was chosen than typically used for in vivo patient scans.

Fourth, this was a specimen study, and further studies evaluating the in vivo detection of microcalcifications with SBCT are necessary.

## 5. Conclusions

Detection of microcalcifications in specimens with SBCT is feasible with a high rate of diagnostic confidence. Sensitivity and specificity of DBT and SBCT are equal when a small voxel size of (0.05 mm)^3^ is used. Further in vivo studies are necessary.

## Figures and Tables

**Table 1:** Sensitivities and specificities for the detection of microcalcifications in 216 segments (prevalence 72.7%).

	DBT	SBCT			*p*–Value (McNemar–Test)					
		Recon1	Recon2	Recon3	DBT vs. Recon1	DBT vs. Recon2	DBT vs. Recon3	Recon1 vs. Recon2	Recon1 vs. Recon3	Recon2 vs. Recon3
reader1										
Sensitivity (95% CI)	94.3% (89.1–97.2)	88.5% (82.2–92.9)	94.9% (89.9–97.6)	92.4% (86.7–95.8)	0.049	1.0	0.508	0.006	0.210	0.125
Specificity (95% CI)	84.7% (72.5–92.4)	94.9% (84.9–98.7)	91.5% (80.6–96.8)	93.2% (82.7–97.8)	0.070	0.388	0.227	0.688	1.0	1.0
reader2										
Sensitivity (95% CI)	93.0% (87.5–96.3)	86.0% (79.3–90.8)	92.4% (86.7–95.8)	93.0% (87.5–96.3)	0.027	1.0	1.0	0.041	0.027	1.0
Specificity (95% CI)	91.5% (80.6–96.8)	94.9% (84.9–98.7)	93.2% (82.7–97.8)	91.5% (80.6–96.8)	0.688	1.0	1.0	1.0	0.688	1.0

**Table 2:** Correct classification of cluster, diffuse microcalcification, and diffuse microcalcifications with a maximum diameter ≤ 0.5 mm.

	DBT	SBCT			*p*-Value (McNemar Test)					
		Recon1	Recon2	Recon3	DBT vs. Recon1	DBT vs. Recon2	DBT vs. Recon3	Recon1 vs. Recon2	Recon1 vs. Recon3	Recon2 vs. Recon3
Cluster										
reader1	79.7%	54.4%	62.0%	59.5%	<0.001	0.001	<0.001	0.210	0.454	0.500
reader2	77.2%	62.0%	75.9%	75.9%	0.017	1	1	0.013	0.019	1
Diffuse Microcalcifications										
reader1	88.9%	81.0%	92.1%	88.9%	0.052	0.289	1	0.001	0.021	0.125
reader2	88.1%	80.2%	85.7%	84.1%	0.078	0.607	0.332	0.170	0.424	0.250
Diffuse Microcalcifications (maximum diameter ≤ 0.5 mm)										
reader1	86.3%	76.5%	90.2%	86.3%	0.052	0.289	1	0.001	0.021	0.125
reader2	85.3%	78.4%	82.4%	80.4%	0.210	0.607	0.332	0.541	0.832	0.625

Prevalence of clusters was 36.6%, diffuse microcalcifications was 58.3%, and diffuse microcalcifications with a maximum diameter ≤ 0.5 mm was 47.2%.

## Data Availability

All data generated and analyzed during this study are included in this published article. Raw data supporting the findings of this study are available from the corresponding author on request.
